# Golimumab as Rescue Therapy for Refractory Immune-Mediated Uveitis: A Three-Center Experience

**DOI:** 10.1155/2014/717598

**Published:** 2014-05-28

**Authors:** Miguel Cordero-Coma, Vanesa Calvo-Río, Alfredo Adán, Ricardo Blanco, Carolina Álvarez-Castro, Marina Mesquida, Sara Calleja, Miguel A. González-Gay, José G. Ruíz de Morales

**Affiliations:** ^1^Department of Ophthalmology, University Hospital of León, 24071 León, Spain; ^2^Uveitis Unit, University Hospital of León, 24071 León, Spain; ^3^Institute of Biomedicine (INBIOMED), University of León, León, Spain; ^4^Department of Rheumatology, University Hospital “Marques de Valdecilla”, IFIMAV, 39008 Santander, Spain; ^5^Department of Ophthalmology, University Hospital “Clínic”, 08036 Barcelona, Spain; ^6^Department of Rheumatology, University Hospital of León, 24071 León, Spain; ^7^Department of Immunology, University Hospital of León, 24071 León, Spain

## Abstract

*Objective*. To evaluate, in three Spanish tertiary referral centres, the short-term safety and efficacy of golimumab (GLM) for treatment of immune-mediated uveitis resistant to previous immunosuppressive therapy.* Methods*. Nonrandomized retrospective interventional case series. Thirteen patients with different types of uveitis that were resistant to treatment with at least 2 previous immunosuppressors were included in this study. All included patients were treated with GLM (50 mg every four weeks) during at least 6 months. Clinical evaluation and treatment-related side effects were assessed at least four times in all included patients. * Results*. Eight men and 5 women (22 affected eyes) with a median age of 30 years (range 20–38) and active immune-mediated uveitides were studied. GLM was used in combination with conventional immunosuppressors in 7 patients (53.8%). GLM therapy achieved complete control of inflammation in 12/13 patients (92.3%) after six months of treatment. There was a statistically significant improvement in mean BCVA (0.60 versus 0.68, *P* = 0.009) and mean 1 mm central retinal thickness (317 versus 261.2 ***μ***, *P* = 0.05) at the six-month endpoint when compared to basal values. No major systemic adverse effects associated with GLM therapy were observed. * Conclusions*. GLM is a new and promising therapeutic option for patients with severe and refractory uveitis.

## 1. Introduction

Corticosteroids remain the mainstay of treatment for the vast majority of patients with immune-mediated uveitis [1]. However, those patients with active inflammation who are intolerant of or unresponsive to steroids require therapy with other immunosuppressive agents trying to prevent the potential sequelae associated with this vision-threatening condition. The off-label use of biologic agents and particularly those blocking tumor necrosis factor-alpha (TNF-*α*) has demonstrated encouraging results when employed for management of patients with immune-mediated uveitis refractory to conventional treatment since their first reported use in 2001 [2]. Potential advantages of these agents when compared with traditional immunosuppressors include a substantial efficacy in recalcitrant cases [3], as well as a lower total immunosuppressive load [4], a rapid clinical effect [5], good safety profile [6], and significant improvement in quality of life [7].

The present evidence shows that infliximab and adalimumab have the highest level of evidence and grade of recommendation, and thus both may be considered as first-line or second-line immunomodulatory agents for treatment of immune-mediated uveitis depending on which systemic immunologic disorder is associated with the intraocular inflammation [6, 8].

Golimumab (GLM) (trade name Simponi), a fully human anti-TNF-*α* monoclonal antibody, was approved by the US Food and Drug Administration in 2009 for the use with methotrexate (MTX) in adults with moderate-to-severe active rheumatoid arthritis (RA) and with or without MTX or other biologic disease-modifying antirheumatic drugs in adults with active psoriatic arthritis (PsA) or active ankylosing spondylitis (AS) [9]. We report the first use of GLM for treatment of noninfectious uveitis in 2011 [10]. Some other studies have addressed the potential use of GLM for treatment of uveitis, mainly associated with rheumatologic conditions [11, 12]. We would like to present the results from three Uveitis Units in Spain when using GLM for treatment of patients with immune-mediated uveitis of various etiologies that had been resistant to several immunosuppressive agents.

## 2. Materials and Methods

*Nonrandomized Retrospective Interventional Case Series. *Patients with different types of active immune-mediated uveitis that had been resistant to local and systemic corticosteroids and at least one additional immunosuppressive agent and who were treated with GLM during at least 6 months were included in this study. All included patients had an associated systemic immune-mediated disease.  Table 1 lists demographic and diagnostic information for the 13 patients who form the basis of this report. In 12/13 patients (92.3%) GLM was at least the second biologic agent used for treatment of uveitis, whereas GLM was used as first-line biologic therapy in one patient (patient 6).  Table 2 shows previous treatment regimens employed for management of uveitis in all included patients.  Table 3 shows the reasons for discontinuation of previous biologic therapy. We defined* primary failure* as an absence of a two-step decrease in level of inflammation (e.g., anterior chamber and/or vitreous cells) or a decrease to grade 0.* Secondary failure* was defined as inflammatory relapse after previous control of inflammation. We classified “control of inflammation” as grade 0 cells in both anterior and posterior segments in addition to absence of other signs of intraocular inflammation (cystoid macular edema (CME) and vasculitis).

All included patients received 50 mg of subcutaneous GLM every four weeks during at least 6 months without modifications during the follow-up. Chest X-ray, tuberculin skin test, and Quantiferon-TB Gold were performed in all patients before treatment. GLM was the only immunomodulatory agent used in six of them. In seven patients, GLM was used alongside previous immunosuppressors, without any dosage modification throughout the study. Topical steroids were used by three patients (patients 6, 8, and 9) at the beginning of the follow-up and were slowly tapered and discontinued after one month in all of them.

Uveitis clinical evaluation was performed at least four times (before treatment and 1, 3, and 6 months after initiation of therapy with GLM) in all included patients. Clinical evaluation included visual acuity (BCVA; best-corrected Snellen VA) and ophthalmic examination. Anterior chamber was graded according to the classification established by the standardisation of uveitis nomenclature; whereas the national eye institute system was adopted for grading vitreous inflammation [13, 14]. Optical coherence tomography (Cirrus HD-OCT, Carl Zeiss Meditec, Dublin, CA, USA) was used before and after treatment in both groups of patients to determine the presence of CME. The 1 mm central retinal thickness was evaluated using the macular cube strategy 512 × 128 in all patients at each study visit. Macular edema was defined as central macular thickness >300 *μ* and/or presence of intraretinal cysts in OCT. Fluorescein angiogram (FA) was performed routinely before and after starting treatment (between 1 and 3 months after initiation of therapy) to determine the presence or absence of retinal angiographic leakage. FA was reviewed for presence or absence of retinal vasculitis and/or CME.

Treatment-related side effects were assessed on each visit with a thorough review of systems and complete blood-cell counts, blood urea nitrogen (BUN) level, creatinine level, and liver function test parameters obtained on an every study visit basis.

Statistical analysis was performed using the software STATISTICA (StatSoft Inc. Tulsa, Oklahoma, USA). Results were expressed as mean ± SD for variables with a normal distribution or as median (25th–75th interquartile range (IQR)) when they were* not normally distributed*. The comparison of continuous variables was performed using the Wilcoxon test.

## 3. Results

Eight men and 5 women (22 affected eyes) with a median age of 30 years (range 20–38) and active immune-mediated uveitides were studied. Uveitis was anterior in 8 patients (61.5%), intermediate in 1 patient (7.6%), and panuveitis in 4 patients (30.7%). All included patients (13/13) received previous treatment with systemic steroids (using intravenous pulses of methyl-prednisolone) in two of them. In addition, four patients (30.7%) received coadjuvant intraocular steroids (2 intravitreal triamcinolone injections and 2 dexamethasone intravitreal implants). About traditional immunosuppressors, all included patients had been treated with methotrexate at any time prior to GLM therapy, whereas cyclosporine A was used in 2 (15.38%), azathioprine in 1 (7.6%), tacrolimus in 1 (7.6%), mycophenolate mofetil in 1 (7.6%), cyclophosphamide in 1 (7.6%), and leflunomide in 1 (7.6%). Only one patient received GLM as first-line biologic therapy, whereas 12 patients received previous treatment with at least one other biologic agent. From the latter, 12 patients received treatment with at least one biologic drug prior to GLM, 7 patients (53.8%) received treatment with at least 2 biologics, 3 patients (23%) received treatment with at least 3 biologics, and 1 patient (7.6%) received treatment with at least 4 biologics. Infliximab (IFX) was used as first biologic agent in 8 patients (61.5%), adalimumab (ADA) in 3 patients, (23%), and etanercept (ETN) in 1 patient (7.6%). Mean time on first biologic treatment was 14.7 months (range 1–50). ADA was used as second biologic agent in 6 patients (46.1%) and ETN in 1 patient (7.6%). Mean time on second biologic agent was 25.5 months (range 15–57). Abatacept was used as third biologic agent in 2 patients (15.3%) and certolizumab in 1 patient (7.6%). Mean time on third biologic agent was 11.6 months (range 2–18). ETN was used as fourth biologic agent in 1 patient (7.6%) during 7 months.

Coadjuvant immunosuppressive therapy was used in 7 of the studied patients including methotrexate (4 patients), azathioprine (1 patient), mycophenolate mofetil (1 patient), and leflunomide (1 patient). Mean time from onset of uveitis to GLM therapy was 97.4 months.

**Figure 1:**
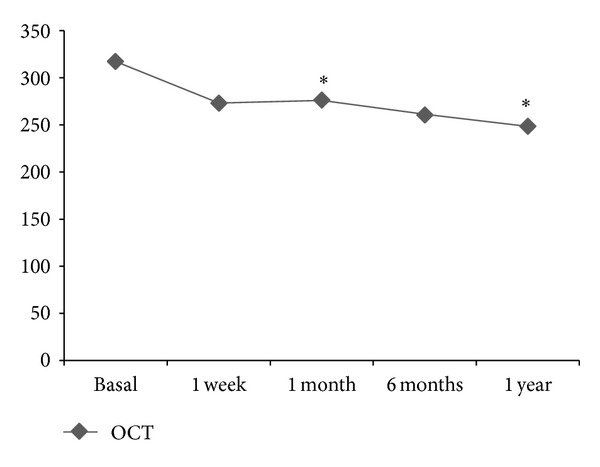
Rapid and maintained improvement of macular thickness (1 mm central retinal thickness, macular cube strategy 512 × 128, and Cirrus-HD OCT) following the onset of Golimumab (data expressed as mean values compared with basal results).

GLM therapy achieved complete control of inflammation in 12/13 patients (92.3%) after six months of treatment. The mean BCVA increased from a basal value (before initiation of GLM) of 0.60 to 0.68 at the six-month endpoint (*P* = 0.009). Only one patient, patient number 12, showed a score of anterior chamber and/or vitreous inflammation different than zero at the six-month endpoint. The mean 1 mm central retinal thickness decreased from a basal value of 317 to 261,2 at the six-month endpoint (*P* = 0.05).  Figure 1 shows changes in mean values of macular thickness (1 mm central thickness) of all included patients over the study period. There was no evidence of active retinal vasculitis before initiation of therapy in any of the included patients. Fluorescein angiogram results did not differ from those observed with OCT regarding the presence of CME.

No major systemic adverse effects were observed. Only a mild and local cutaneous reaction was recorded in two patients (patients 5 and 9) among all included patients over the entire study period.

## 4. Discussion

These results suggest that GLM is well tolerated and is associated with control of inflammation in 92,3% of a heterogeneous group of immune-mediated uveitis patients resistant to traditional therapy and other biologic agents. The use of GLM is also associated with short-term improvement in mean values of BCVA and decrease of mean values of central retinal thickness. Despite the evident limitations of this study, including its retrospective design, lack of a control group, short follow-up, and limited number of patients, the results suggest that further evaluation of this modality is appropriate.

TNF-*α* is recognized as one of the main inflammatory cytokines involved in the pathogenesis of immune-mediated uveitis [14–16]. This ubiquitous cytokine plays a key role in initiating and maintaining the inflammatory processes by orchestrating leukocyte infiltration, dendritic cell maturation, and macrophage activation and driving T-helper lymphocytes' response [17]. Therapy with two TNF-*α* inhibitors, infliximab and adalimumab, has been proven to be effective for treatment of immune-mediated uveitis with considerable levels of recommendation and evidence [6, 8]. The clinical efficacy of other TNF-*α* blockers such as GLM needs to be demonstrated and thus this drug is considered only as an alternative to those patients who have failed to respond to first-line TNF-*α* inhibitors. However, it is necessary to emphasize that not all patients respond to their first anti-TNF agent, and so it is clearly useful to have a range of effective therapeutic options to treat those patients with severe and refractory immune-mediated uveitis. In this setting, the role of GLM in the noninfectious uveitis treatment algorithm needs to be further studied.

A potential limitation of these results could be related to the inclusion of less severe uveitis considering that we include 8 patients with anterior uveitis. However, anterior uveitis in these patients was associated with juvenile idiopathic arthritis (patients 5, 9, 10, and 11) and HLA-B27 + haplotype (patients 1, 4, 6, and 8). Despite recent therapeutic progress, JIA-associated uveitis has a severe course and the potential for long-term complications, including blindness [18]. HLA-B27 positive-associated anterior uveitis is associated with a substantially higher incidence of ocular complications and has a much worse prognosis when compared with HLA-B27 negative-associated anterior uveitis [19]. Moreover, some of the patients with anterior uveitis (patients 2, 9, and 11) had concomitant macular edema, the most vision-threatening complication associated with uveitis [20, 21]. In this setting, we cannot consider all anterior uveitis as “benign” entities. The seven cases that were included in our paper were severe and vision-threatening cases refractive to conventional and nonconventional treatment for such conditions.

Of note, patients included in the present study had a severe inflammatory condition which was resistant to several treatment-regimens inflammatory conditions. The positive response observed in almost all included patients needs to be analysed in this mentioned difficult clinical setting. This* inclusion bias* in addition to the short follow-up may have influenced the limited improvement in visual acuity observed in our study considering the long and severe course of intraocular inflammation in our patients.

Interestingly we observed a significant improvement in central retinal thickness. TNF-*α* is one of the inflammatory cytokines that upregulates intraocular production of VEGF [22, 23], which plays a crucial role in the pathogenesis of CME [24]. We have previously reported how another TNF-*α* blocker, adalimumab, induces a reduction in plasma VEGF levels when employed for treatment of immune-mediated uveitis, which may correlate with clinical improvement [25]. In our study GLM also demonstrated a beneficial effect on CME thus strengthening the idea of a comparable efficacy with first-line TNF-*α* blockers.

Previous reports on the use of GLM for treatment of uveitis have focused on those patients with uveitis secondary to Behçet disease, juvenile idiopathic arthritis, and/or HLA-B27 + haplotype. To the best of our knowledge, this is the first report of GLM employed in the treatment of uveitis associated with sarcoidosis, Vogt-Koyanagi-Harada disease, and/or psoriatic arthritis. The increasing potential treatment indications for GLM may be of high interest in the therapeutical decision making of chronic uveitis patients.

The main aim of this study is to show these encouraging results on the use of GLM for treatment of immune-mediated uveitis. Although these results are preliminary, further studies including a higher and more homogeneous group of patients are warranted.

## Figures and Tables

**Table 1:** Demographic and diagnostic information of all included patients.

Patient number	Age	Sex	Affected eye	Associated disease	Location of uveitis	AC infl (SUN)	Vitr infl (SUN)	Macular edema^1^
1	36	M	OD	Psoriatic arthritis	Anterior	Active	Inactive	No
2	20	F	OU	Sarcoidosis	Panuveitis	Active	Inactive	Yes
3	27	M	OU	Sarcoidosis	Intermediate	Inactive	Active	No
4	31	F	OD	Psoriatic arthritis	Anterior	Active	Inactive	No
5	34	M	OD	JIA	Anterior	Active	Inactive	No
6	37	M	OS	Axial SpA	Anterior	Active	Active	Yes
7	22	M	OU	VKH	Panuveitis	Active	Active	Yes
8	32	M	OU	Axial SpA	Anterior	Active	Inactive	Yes
9	21	F	OU	JIA	Anterior	Active	Inactive	Yes
10	23	F	OU	JIA	Anterior	Active	Inactive	Yes
11	24	F	OU	JIA	Anterior	Active	Inactive	No
12	38	M	OU	Behçet	Panuveitis	Active	Active	Yes
13	30	M	OU	Behçet	Panuveitis	Inactive	Active	Yes

M: male; F: female; OD: right eye; OS: left eye; OU: both eyes; JIA: Juvenile idiopathic arthritis; VKH: Vogt-Koyanagi-Harada syndrome; Ac infl (SUN): anterior chamber inflammation base on *Standardization of uveitis nomenclature criteria*. (Ref.) Vitr infl (SUN): vitreous inflammation base on *Standardization of uveitis nomenclature criteria*. (Ref.).

^1^Macular edema was defined as central macular thickness >300 *μ* and/or presence of intraretinal cysts in optical coherence tomography (Cirrus HD-OCT, Carl Zeiss Meditec, Dublin, CA, USA). The 1 mm central retinal thickness was evaluated using the macular cube strategy 512 × 128.

**Table 2:** Previous immunosuppressive therapies in all included patients.

Previous treatment	
CsA	2
AZA	1
MTX	11
Bolus of methylprednisolone i.v.	2
Biologic therapy	
First biologic drug used:	
IFX	8
ADA	3
ETN	1
Monotherapy/combined treatment	4/9
Second biologic drug used	
ADA	6
ETN	1
Monotherapy/combined treatment	1/6
Third biologic drug used	
Certolizumab	1
Abatacept	2
Monotherapy/combined treatment	0/3
Fourth biologic drug used	
ETN	1
Monotherapy/combined treatment	0/1

CsA: cyclosporine A; AZA: azathioprine; MTX: methotrexate; IFX: infliximab; ADA: adalimumab; ETN: etanercept.

**Table 3:** Reasons for discontinuation of previous biologic therapy.

First biologic drug used	
Primary failure	5
Secondary failure	2
Toxicity	3
Second biologic drug used	
Primary failure	3
Secondary failure	4
Toxicity	0
Third biologic drug used	
Primary failure	2
Secondary failure	1
Toxicity	0
Fourth biologic drug used	
Primary failure	1
Secondary failure	0
Toxicity	0
